# Adsorption, Desorption, Resorption

**DOI:** 10.6028/jres.067A.061

**Published:** 1963-12-01

**Authors:** William V. Loebenstein

## Abstract

The complete characterization of batch adsorption from solution, desorption, and related phenomena have been interpreted in the light of a general equation. The forward and reverse adsorption-rate constants and the adsorptive capacity comprise the only parameters. Where adsorption alone is of importance and the desorption-rate constant can be neglected, a simplified form of the theory results in a special equation which may suffice for most adsorption purposes. In either case, the characteristic parameters are determinable from the data and serve as criteria for comparing similar systems. The theory has been confirmed by the data of various investigators taken from the literature. The parameters derived from column adsorption are in agreement with the corresponding batch-derived parameters. The limitations as well as the capabilities of the theory are presented; but even where deviations from the assumed model exist, the results are useful.

## 1. Introduction

In earlier publications [1, 2]^1^ the basis was established for characterizing adsorption from solution in terms of just two parameters, namely; the adsorptive capacity per gram of adsorbent, *q*_0_, and the specific adsorption-rate constant, *k*_1_. The values of corresponding parameters derived from batch and from column adsorption were shown to be in substantial agreement with one another, respectively. The two-parameter equations are based on the assumptions that the adsorption step is monomolecular, irreversible, and rate controlling. Furthermore, the assumption of a uniform surface is implicit in the theoretical treatment, since the differential equations used in the derivations are essentially of the Langmuir type. Although these requirements may not be completely applicable in any given instance, the equations are still useful insofar as they provide an approximation of the characteristic parameters which may not be obtainable by other means. The present paper deals, to a considerable extent, with the treatment of data which fall in this category.

In the more general case where reversibility must be reckoned with, but otherwise subject to the same limitations mentioned, a three-parameter batch adsorption equation has been derived [2] which includes the desorption rate constant *k*_2_. For the first time a means is afforded for predicting desorption into solvent as well as adsorption from solution with equal facility. Perhaps even more interesting is the phenomenon of resumed sorption or “resorption” following the interruption of an initial adsorption or desorption step. Should an initial adsorption process, for example, be interrupted and the resumption preceded by a deliberate and sufficient lowering of the concentration, the theory predicts a change to desorption in agreement with experience.

## 2. Two-Parameter Batch Equation

The two-parameter batch adsorption equation previously derived by integrating the irreversible rate equation was shown to be:

$$\frac{q}{q_{0}}=\frac{1-e^{-\left(\frac{Wq_{0}}{Vc_{0}}-1\right)c_{0}k_{1}t}}{\frac{Wq_{0}}{Vc_{0}}-e^{-\left(\frac{Wq_{0}}{Vc_{0}}-1\right)c_{0}k_{1}t}}$$ (1)

where:

*q*: = the amount of solute adsorbed per gram of the adsorbent at any time, *t*;
*q*_0_: = the maximum value *q* would have if all of the adsorption sites were filled;
*c*: = the instantaneous solute concentration whose initial value is *c*_0_;
*W*: = the weight of the adsorbent;
*V*: = the volume of the solution; and
*k*_1_: = the specific adsorption-rate constant.

Methods are available for obtaining values of the parameters *q_0_* and *k*_1_ which give an optimum fit of eq (1) to the experimental data in the general case where *W, V, c*_0_, and *t* may all vary from point to point. At best, however, they are cumbersome, and shortcut methods will certainly be preferred wherever they can be used.

One such method was worked out [2] for the special case where both *W/V* and *c*_0_ are held constant. Under these conditions *q* values, *q*_1_ and *q*_2_, are determined corresponding to times *t*_1_ and *t*_2_, respectively, such that *t*_2_=2*t*_1_. It was then shown that:

$$q_{0}=\frac{q_{1}^{2}\left[q_{2}\left(\frac{W}{Vc_{0}}\right)-1\right]}{q_{1}^{2}\left(\frac{W}{Vc_{0}}\right)-2q_{1}+q_{2}}$$ (2)

and

$$k_{1}=\frac{\ln\;\left[\frac{q_{0}-q_{1}\left(\frac{Wq_{0}}{Vc_{0}}\right)}{q_{0}-q_{1}}\right]}{c_{0}t_{1}\left(1-\frac{Wq_{0}}{Vc_{0}}\right)}.$$ (3)

Use was made of eqs (2) and (3) in an example taken from published data of Dryden and Kay [3] for the adsorption of aqueous acetic acid on a steam-activated coconut carbon. Good agreement was obtained from three independent determinations of *q*_0_ and *k*_1_. This agreement would not have resulted if the neglected desorption rate constant had been appreciable.

Another special case whose derivation and solution are very similar to the aforementioned method occurs when *t* is constant providing that a second condition is satisfied. This is the requirement that two values of *q* can be found, say, *q*_1_[(*W/V*)_1_, (*c*_0_)_1_] and *q*_2_[(*W/V*)_2_, (*c*_0_)_2_] such that (*W/V*)_2_=2(*W/V*)_1_ and (*c*_0_)_2_=2(*c*_0_)_1_. Under these conditions the recurring quantity $\frac{Wq_{0}}{Vc_{0}}$ which appears so prominently in eq (1) remains unchanged and it follows that

$$q_{0}=\frac{q_{1}^{2}\left[q_{2}{\left(\frac{W}{Vc_{0}}\right)}_{1}-1\right]}{q_{1}^{2}{\left(\frac{W}{Vc_{0}}\right)}_{1}-2q_{1}+q_{2}}$$ (4)

and

$$k_{1}=\frac{\ln\;\left[\frac{q_{0}-q_{1}{\left(\frac{Wq_{0}}{Vc_{0}}\right)}_{1}}{q_{0}-q_{1}}\right]}{{\left(c_{0}\right)}_{1}t\;{\left(1-\frac{Wq_{0}}{Vc_{0}}\right)}_{1}}.$$ (5)

## 3. Three-Parameter Equation for Batch Adsorption

It has also been shown [2] that where it is desired to retain the desorption rate constant, *k*_2_, in batch adsorption, the integrated equation takes the form:

$$\frac{(M-N)-q}{(M+N)-q}=\frac{M-N}{M+N}e^{-2\left(\frac{W}{V}\right)Nk_{1}t}$$ (6)

where *M* and *N* are defined as:

$$M=\frac{V}{2W}\left[\frac{k_{2}}{k_{1}}+c_{0}\left(1+\frac{Wq_{0}}{Vc_{0}}\right)\right]$$ (7)

$$M^{2}-N^{2}=q_{0}\left(\frac{Vc_{0}}{W}\right).$$ (8)

Here again the parameters *q*_0_, *k*_1_, and *k*_2_ can be readily estimated from a single batch adsorption experiment in the special case where *q* is determined as a function of *t.* The solution concentration is measured when *t* takes on the values^2^: *i*, 2*i, j*, and 2*j.* By using the same type of reasoning which led to eqs (2) and (3) from eq (1), it is possible to show from eq (6) that

$$2q_{i}^{2}M-(2q_{i}-q_{2i})(M^{2}-N^{2})=q_{i}^{2}q_{2i}.$$ (9)

Equation (10) can be written by inspection,

$$2q_{j}^{2}M-(2q_{j}-q_{2j})(M^{2}-N^{2})=q_{j}^{2}q_{2j}$$ (10)

since it differs from eq (9) only in the subscripts. Equations (9) and (10) constitute a set of simultaneous equations in two unknowns, *M* and (M^2^*−N^2^*), for which the solution is easily obtained. Once these quantities have been found, (*M*+*N*) and (*M−N*) can readily be solved for use in eq (6). Back substitution of one experimental point is sufficient for the determination of *k*_1_. Equation (6) can then be used to predict *q* for all values of *t.*

In the event that only the value of *q*_0_ is desired in a given instance, it is only necessary to determine (*M*^2^*−N*^2^) from eqs (9) and (10) for use in eq (8). The quantity (*M*^2^−*N*^2^) is given (according to Cramer’s rule) by the ratio:

$$\frac{\begin{array}{cc} |\begin{array}{l} 2q_{i}^{2}\\ 2q_{j}^{2} \end{array} & \begin{array}{l} q_{i}^{2}q_{2i}\\ q_{j}^{2}q_{2j} \end{array}| \end{array}}{\begin{array}{cc} |\begin{array}{l} 2q_{i}^{2}\\ 2q_{j}^{2} \end{array} & \begin{array}{l} -(2q_{i}-q_{2i})\\ -(2q_{j}-q_{2j}) \end{array}|. \end{array}}$$

Consider the example (Dryden and Kay’s Run #201) already cited for the two-parameter equation for purposes of illustration and comparison. The amounts of acetic acid adsorbed per gram of charcoal are repeated in table 1 corresponding to the measured solution concentrations. If, arbitrarily, *i* and *j* are taken as 10 min and 15 min, respectively, it follows that

$$\begin{array}{l} q_{i}=0.3533\;q_{j}=0.4033\\ q_{2i}=0.4667\;q_{2j}=0.5033. \end{array}$$

Substitution of these values in eqs (9) and (10) results in *M^2^*−*N*^2^ = 0.63965 so from eq (8),

$$q_{0}=0.627\;\text{meq}\cdot g^{-1}.$$

To continue with the illustration, *M*=0.848. Thus, *M−N*=0.566 and *M+N*=1.130. By substituting *q*_0_ and *M* back into eq (7), it can be verified that *k*_2_/*k*_1_=0.00147. This confirms that the desorption rate constant is, indeed, very small compared with *k*_1_. One of the adsorption points, say, *q*=0.3533 for *t*=10 may now be substituted back in eq (6) to solve for *k*_1_. The two rate constants are

$$k_{1}=3.57\;\text{ml}\cdot {\text{meq}}^{-1}\cdot \min^{-1}\;k_{2}=0.00524\;\min^{-1}$$

and eq (6), in this particular instance, reduces to:

$$\frac{0.566-q}{1.130-q}=0.501e^{-0.0604t}.$$

It should be pointed out that the value of 0.627 obtained here for *q*_0_ is about 10 percent higher than the corresponding value previously cited [2] for the simpler case where irreversibility was assumed. This (present) value is, moreover, consistent with independent *q*_0_ determinations of 0.666 and 0.641 for combinations of *i*=15 with *j*=2*i*=30 and for *i*=10 with *j*=30, respectively. The value of 3.57 for *k*_1_ compares well with the corresponding value from the two-parameter equation.

## 4. Application of Batch Adsorption Theory for Different Values of c_0_

A 1944 publication by W. G. Burgers [4] afforded the opportunity to test the applicability of eq (1) and/or eq (6) to the case where the initial concentration differed from batch to batch.

Acetic acid was adsorbed on pulverized “Norit” charcoal at 25 °C with continuous agitation for periods of 2 hr. The volume of solution was held constant at 50 cm^3^ while the weight of the carbon and the initial concentration of acid were varied in accordance with table 2 which also lists the corresponding values for the amounts of acetic acid adsorbed per gram. The experimental data of table 2 are reproduced directly from the first two columns of Burgers’ *Tableau I*, *Tableau II, et Tableau III.*

In the estimation of *q*_0_ and *k*_1_ through the application of eqs (4) and (5), the value of 2.024 mM·g^−1^ was selected for *q*_2_ corresponding to (*W/V*)_2_=0.04: g·cm^−3^ (i.e., *W*_2_=2.0 g) and the corresponding value of (*c*_0_)_2_ was, consequently, 0.3779 mM·cm^−3^. No measurement was available for *q*_1_ corresponding to 0.1890 mM·cm^−3^ for (*c*_0_)_1_ and (*W/V*)_1_=0.02 g·cm^−3^. However, a conventional plot of the data by Burgers showed very little scatter of the points, hence an interpolation was made between the close neighboring values resulting in *q*_1_≈1.683 mM·g^−1^.

Equation (4) yielded *q*_0_≈2.1 and this was retained for use with the three-parameter equation. While a somewhat lower value than 6.0 was obtained from eq (5) for *k*_1_ this value was tested along with an estimate for *k*_2_ such that *k*_2_,/*k*_1_≈0.02.

Although lengthy optimizing techniques are available for obtaining a “best” fit of the parameters in eqs (6–8), no improvement was sought in this application. The purpose was to show that the agreement is reasonably close between calculated and experimental values of *q* despite the use of these rounded off first estimates of *q*_0_, *k*_1_, and *k*_2_. This comparison is shown in the last two columns of table 3. The first two columns identify the points, while the intermediate columns list the values computed for the component parts of eqs (6–8) for each determination.

## 5. Analysis and Comparison of Parameters from Column and Batch Adsorption

An example has already been given in an earlier paper [2] of the application of the present adsorption theory to a batch adsorption run described by Dryden and Kay [3]. That run was part of a wealth of experimental data contained in the Ph.D. Thesis of C. E. Dryden [5]. An interpretation of the results of that data in the light of the present theory can now be readily made. Dry den’s experiments consisted of some 20-odd column adsorptions (static bed experiments), a somewhat shorter series of column desorptions, nearly 20 batch adsorptions, and 7 batch desorptions. All runs were carried out at 30 °C using acetic acid together with a steam-activated coconut carbon.

In the column adsorption experiments, a 4-fold variation in column height was used from run to run. Extreme values of volume-flow rate varied over a 20-fold range. Four U.S. Standard sieve sizes of charcoal were used ranging from (8 on 10) to (40 on 60). Two levels of initial acetic acid concentration were employed; namely, 0.10 *N* and 0.31 *N.*

In the batch adsorption experiments, the rate of agitation was varied from 0 to 400 rpm of magnetic stirring. The sieve fractions range from (8 on 10) to (80 on 100) in five steps. Water-wetted charcoal was compared with the customary initially dry material. The two levels of initial acetic acid concentration used were 0.03 *N* and 0.10 *N.*

### 5.1 Parameters Derived from Column Adsorption Runs

For each of the column adsorption experiments a semilogarithmic plot of *(c*_0_*/c*) — 1 against throughput, *y*, was made in order to determine the characteristic parameters, *q*_0_ and *k*_1_. This is in accordance with the equation

$$\ln\;\left(\frac{c_{0}}{c}-1\right)=\frac{k_{1}q_{0}x}{\dot{V}}-\frac{k_{1}c_{0}y}{\dot{V}}$$ (11)

which had been derived and tested in earlier work [1]. In eq (11)*V* is the volume-velocity; *x* is the mass of adsorbent upstream from the point at which effluent is collected; and *y* is the throughput or cumulative volume of solution which has passed that point since the start.

The initial (low throughput) points of the curves were not used in fitting to the linear requirement of eq (11). The substitution of solution for the water used to settle the columns is not, strictly speaking, a piston-displacement-like process. Consequently, the early values of *c* should be abnormally low resulting in initially high values for *(c*_0_*/c) —* 1. This phenomenon has been observed in other work [2], as well. The values of the parameters *q*_0_ and *k*_1_ consequently determined from the final points of each plot are shown in table 4 along with the conditions applicable to each run.

#### a. Agreement With Theory

The most significant result which is immediately evident from table 4 is the degree of agreement among the computed values of *q*_0_. The spread in sieve sizes corresponds to a range in mean particle diameter from about 360 to nearly 2200 microns. The initial concentration varies over three-fold. The velocity of flow ranges from 3.32 ml·min**^−1^** to 64.6 ml·min**^−1^**. The weight of adsorbent varies between about 15.5 g and 60 g. Yet, notwithstanding the interplay of these factors, for the results of the 18 runs at which *c*_0_=0.10, there yielded a mean *q*_0_ value of 1.264 meq·g**^−1^** with a standard deviation of 0.263.

#### b. Anomalous Effects

A closer scrutiny of table 4 discloses several interesting facts. The excellent data make it possible to discern “second order” effects which cannot be interpreted in the light of the present simplified theory.

A comparison of Run #55 with #60, of #83 with #91, and of #84 with #92 suggest that the effect of a three-fold increase in the initial concentration, *c*_0_, other things being equal, resulted in an increase in *q*_0_ of about one-and-one-half-fold. This can readily be explained as a departure from idealized Langmuir behavior. The Langmuir model implies a uniform surface. If this is only approximated, then the number of adsorbing sites (a measure of *q*_0_) which could be capable of participating in the case of a much greater initial concentration of solute would include some portions of the surface requiring higher activation energies. If, moreover, adsorption proceeded more slowly at these latter sites, it would result in a lower overall *k*_1_ value.

Another “second order” effect appears to be present in considering flow rates. Other things being equal, an increase in velocity results in a lower value for *q*_0_ and a higher value for *k*_1_ as can be seen from table 4. This behavior would be expected based on diffusion considerations which have been entirely neglected in the development of the present simplified theory. The greater the flow rate becomes, the more difficult it is to insure equal access of solute to all of the absorbing sites. Thus, the computed value of *q*_0_ based upon experiments at the higher flow rates would be underestimated. This reasoning is consistent with overestimated values for *k*_1_.

**Figure 1:**
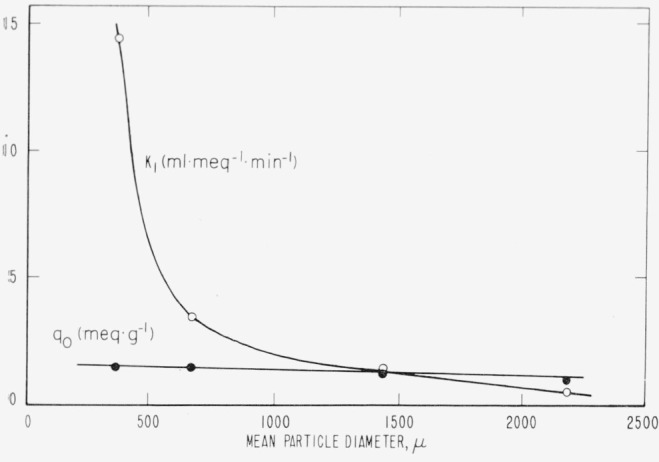
Dependence of parameters on particle size. The parameters were computed from column adsorption. Each point on both curves represents the average of three to five individual determinations. Runs with essentially the same initial concentrations were used, namely, 0.10 meq·ml^−1^

The variation in sieve size (particle diameter) has almost a negligible effect upon *q*_0_ although its influence on *k*_1_ is quite pronounced. These comparisons are portrayed quite strikingly in figure 1. Phenomena such as the very small dependence of *q*_0_ on particle size are of particular interest in confirming the physical significance of the derived parameters. Capacity for adsorption, like surface area, is a quantity measurable only at the molecular level. The process of subdividing a highly porous particle creates very little additional surface not already accessible to a molecule.

### 5.2 Parameters Derived From Batch Adsorption Runs

The quantities *q*_0_ and *k*_1_ were calculated from the data of each of the batch adsorption runs reported. In general, eqs (2) and (3) were employed for this purpose, the fact having been established that the desorption rate constant *k*_2_ was negligibly small compared with *k*_1_.

As an example to illustrate the procedure, the data and calculations for Batch #221 are typical. These data are given in table 5. The 30 and 60-min points corresponding to 0.245 and 0. 258 meq·g^−1^ for *q*_1_ and *q*_2_, respectively, were selected for use with eqs (2) and (3) to determine *q*_0_ and *k*_1_:

$$q_{0}=\frac{0.060[0.258(3.268)-1]}{0.060(3.268)-0.490+0.258}=0.262\;\text{meq}\cdot g^{-1}$$

$$k_{1}=\frac{\ln\;\left[\frac{0.262-0.245(0.8562)}{0.262-0.245}\right]}{\left(0.0306)(30)(1-0.8562\right)}=8.50\text{ml}\cdot {\text{meq}}^{-1}\cdot \min^{-1}.$$

If one had chosen the 15 and 30-min points instead, the computation for *q*_0_ would have been:

$$q_{0}=\frac{0.0493[0.245(3.268)-1]}{0.0493(3.268)-0.444+0.245}=0.259\;\text{meq}\cdot g^{-1}$$

In general, the greater time intervals were consistently chosen and were considered most reliable.

While it is possible to utilize eqs (9) and (10) for determining the parameters in accordance with the general adsorption equation as previously illustrated, this practice is only required when the desorption rate constant, *k*_2_, is appreciable relative to *k*_1_. The simpler method shown here will be preferred wherever it can be used.

In like manner, values for *q*_0_ and *k*_1_ were determined for all of the batch experiments. These results are grouped so as to bring out most effectively the possible influence of each of the factors studied such as rate of stirring, sieve size, etc.

#### a. Initially Dry Versus Prewetted Adsorbent

A few batch runs were described in Dryden’s Thesis [5] in which the adsorbent had been presoaked in water prior to contact with the acetic acid solution. It was hoped to ascertain whether presoaking had any effect upon the adsorption. It now appears clear, in light of the present theory, that the prewetted adsorbent gave rise to *q*_0_ and *k*_1_ values which fell in line with those from the initially dry adsorbent. These results are shown in table 6.

The volume of the solutions were 100 ml and the initial concentration of acetic acid was 0.03 meq·ml^−1^. There was a slight dilution effect caused by the water contained in the presoaked samples as reflected by the increase in *V* and decrease in *c*_0_. However, this was limited to 10 percent in all cases and is seen to have a minor effect at most compared with the influence of changes in *W.*

The values of *q*_0_ and of *k*_1_, of course, should be constant if the ideal conditions assumed in the derivation of the theory were closely approximated. The observed trend, attributable to the increase in the *W/V* ratio, is undoubtedly caused by a departure from these conditions.

#### b. Effect of *W/V*

The effect of *W/V* is equivalent to the effect of *W* in this work since *V* was held constant at 100 ml. (Runs #236 and #238, alone, had slightly higher values because of presoaking.) Tables 6 and 7 show the results of increasing adsorbent weight. The essential distinction between the two tables is the sieve sizes although these differences are not pronounced because the sizes are close together. A more searching comparison of the effect of sieve sizes is taken up later. The important point here is that the value determined for the parameter *q*_0_ decreases with increasing *W.* Both tables confirm that a 10-fold change in *W* results in about a 3-fold change in *q*_0_. The parameter *k*_1_ is also affected by a change in *W.* As *q*_0_ decreases, *k*_1_ increases. It is about twice as sensitive as *q*_0_, moreover, to changes in *W*.

#### c. Effect of Stirring Rates

The rate of stirring was varied in three steps from 0 to 400 rpm within each of two sets of experiments. The sets differed from one another in regard to sieve size. The results are shown in tables 8-a and 8-b. Within each set there is no apparent correlation of parameters with stirring rate. The observed spread in values of *q*_0_ are entirely within experimental error. The same is true for *k*_1_, except perhaps for the unusually high value obtained in Run #209. No reason nan be found for this singular anomaly.

#### d. Effect of Initial Concentration

The influence of *c*_0_ on the results of batch adsorption are strikingly similar to those for column. Although only two batch runs were made at *c*_0_=0.10, ’ these are sufficient for comparison purposes. Tables 9-a and 9-b compare these batches (Runs #202 and #215) with other batch runs which differed only with respect to initial concentration. The *q*_0_ values of 1.425 and 1.201 meq·g^−1^ obtained in Runs #202 and #215, respectively, compare well with 1.264 meq·g^−1^, the average of the 18 column runs previously computed for the same initial concentration. While *k*_1_ seems to be more sensitive to variations in conditions than does *q*_0_, its magnitude is also consistent with the corresponding column results.

It is interesting that a three-fold increase in initial concentration from 0.03 to 0.10 meq·ml^−1^ (as seen in tables 9-a and 9-b) resulted in nearly a three-fold increase in *q*_0_. However, at higher initial concentrations the effect was much less pronounced. This can be seen from table 4 by comparing Run #55 with Run #60; Run #83 with Run #91; and Run #84 with Run #92. In each of these comparisons where factors other than *c*_0_ were essentially constant, the initial concentration increased from 0.10 to 0.31 meq·ml^−1^; yet the increase in *q*_0_ was limited to about 50 percent.

#### e. Effect of Sieve Size

The influence of particle size on the parameters derived from the batch adsorption experiments confirms the findings of the column runs. Very little, if any, change in *q*_0_ is evident from table 10-b, although the sieve size ranges in five steps from (8 on 10) through (80 on 100), other factors being constant. At the same time, however, the accompanying value of *k*_1_ increased markedly with decreasing particle size. Tables 10-a, 10-c, and 10-d show the same lack of dependency of *q*_0_ although only two runs were available for comparison in each case.

In a preceding paper [2], the adsorbent involved was a service bone char which had been subjected to numerous cycles of adsorption, partial desorption, drying, and kilning. Its prior history may have been reflected in its dependence of *q*_0_ on sieve size in contrast with the present study. This very point was discussed in some detail at that time.

## 6. Adsorption—Desorption—Resorption

Much has been written in the preceding sections of this paper regarding the limitations of the present theory. Examples have been given and comparisons made showing the extent of departure from ideal conformity with the model assumed, although plausible explanations were offered for most of the observed discrepancies. Despite these shortcomings, the theory has much to recommend it including applications which have not heretofore been discussed. One such application is in desorption. It is clear, of course, that the simplified two-parameter equation cannot be used in this application, since it neglects entirely the desorption rate constant, *k*_2_. Furthermore, it would be extremely desirable to be able to use only one equation for both adsorption and desorption.

The difference between the two processes should be reflected only in the boundary conditions. In the derivation of the adsorption equation the initial conditions required all of the adsorbable species to be in the solution phase. Conversely, for desorption the adsorbable species initially would be entirely in the adsorbed phase. To proceed one step further, it might be stipulated that both adsorption and desorption should be considered, from this point of view, as special cases of an initial condition where some of the adsorbable species may exist in solution while the remainder is adsorbed. The process which would subsequently take place might either be adsorption or desorption, depending upon the levels of the interrelated variables. These various concepts may be reconciled by use of the term “resorption” to define this resumed sorption process.

In the original derivation of eq (6), the quantity *c*_0_ was defined as the concentration of the adsorbable solute before any adsorption had taken place. For the general case (applicable as well in the original case), *c*_0_ should be redefined as follows:

$$c_{0}=\text{the concentration that would exist at any time if all of the adsorbable species were assumed to be in the solution phase}.$$

Two new symbols can now be defined as c_I_ and *q*_I_ to correspond to the concentration and the amount adsorbed per gram, respectively, which exist at the onset of a sorption process.

Since the conservation equation holds under all conditions, it follows here that

$$c_{0}=\frac{W}{V}q_{I}+c_{I}$$ (12)

and the general form of the integrated equation becomes

$$\frac{(M-N)-q}{(M+N)-q}=\frac{(M-N)-q_{I}}{(M+N)-q_{I}}e^{-2\left(\frac{W}{V}\right)Nk_{1}t}$$ (13)

while *M* and *M*^2^*−N*^2^ retain their definitions as given by eqs (7) and (8), respectively.

It is seen that the only difference between eq (13) and eq (6) is the appearance of *q*_I_ in numerator and! denominator of the coefficient of *e.* Reference to eq (12) confirms that for an adsorption process *q*_I_=0 and *c*_0_=*c*_I_. Under these conditions eq (13) reduces to eq (6) as a special case. For a desorption process where the adsorbent containing adsorbate is added to pure solvent, *c*_I_ vanishes and eq (12) shows that *Vc*_0_*/W=q*_I_. Obviously, in any case, it is always true that *q*^0^*≥q*_I_. Since *q*_I_ is different from zero in this instance (desorption), eq (13) would apply.

### 6.1. Desorption

The consequence of subtracting *q*_I_ from the numerator and denominator of the coefficient of *e* in eq (13) can impart a negative value to this factor which immediately identifies the process as one of desorption. It is instructive to consider the batch desorption data of table 11 as an example of the use of eq (13) in this capacity. The table contains the data collected by Dryden in Run #224.
Amount of acetic acid preadsorbed on the charcoal7.26 meqWeight of wet charcoal8.036 gWeight of dry charcoal (*W*)5.221 gDifference (assumed to be excess water)2.82 mlWater added100.00 mlTotal water present, (*V*)102.82 ml

$$q_{I}=\frac{7.26}{5.221}=1.391\;\text{meq}\cdot g^{-1};\;c_{0}=\frac{7.26}{102.82}=0.07061\;\text{meq}\cdot {\text{ml}}^{-1}.$$

While it is possible, analytically, to solve the desorption equation using a method based on the same principles as in the case of adsorption, it is considerably more involved. It is extremely sensitive both to the accuracy of each of the three or four measured points used, as well as to the slightest departure from the assumed model. For these reasons the usefulness of this method for determining the parameters is purely academic.

For the example used in this illustration, a reasonably fair agreement with the desorption data can be obtained using the approximate values:

$$\begin{array}{l} q_{0}=2.0\;\text{meq}\cdot g^{-1}\\ k_{1}=1.0\;\text{ml}\cdot {\text{meq}}^{-1}\cdot {\text{min}}^{-1}\\ k_{2}=0.045\;{\text{min}}^{-1}. \end{array}$$

It is noteworthy that the magnitude of each of these parameters is consistent with corresponding values derived from adsorption. Equation (13) can now be evaluated. It is first determined by eq (7) that *M*=2.138 under the conditions of the experiment. Next, it is ascertained by use of eq (8) that *N*= 1.338. The coefficient of *t* in the exponent of eq (13) can now be determined as well as the factor:

$$\frac{(M-N)-q_{I}}{(M+N)-q_{I}}.$$

Accordingly, the desorption equation reduces to:

$$\frac{0.800-q}{3.476-q}=-0.283\;e^{-0.136t}.$$

It can be seen that as *t* becomes large, the right side of the equation approaches zero. Therefore, the limiting value of *q* must be 0.800 in agreement with table 11. At the other extreme the value of *q* predicted for 1 min is 1.33 compared with 1.190 as seen from the table. The remaining desorption experiments reported by Dryden [5] yield results in substantial agreement with the example given here.

### 6.2. Resorption

The remarkable versatility of eq (13) cannot be fully appreciated until some examples of resorption are considered. Fortunately, it is not necessary to redesign additional experiments to illustrate these applications.

For the first example, consider desorption Run #224 just discussed. The desorption equation predicts *q*=0.81 meq·g^−1^ for *t*=30 min. Suppose that after desorption had progressed for 10 min, the process were halted by physically separating the adsorbent from solution for an indefinite period of time. Ultimately, adsorbent and solution could be recombined, thus permitting the desorption process to be resumed. Reference to table 11 discloses that when *t*=10 min, 0.912 meq·g^−1^ is the observed value of *q* which, consequently, would become the new value for *q*_I_ in the resorption process. Neither *M* nor *N* would change, since the weight, volume, concentration, etc., were not altered. The new coefficient of the exponential in eq (13) would be:

$$\frac{0.800-0.912}{3.476-0.912}=-0.0437$$

while the only change in the exponent, itself, would be the substitution of (*t*—10) for *t*. Almost by inspection, therefore, the new resorption equation could be written :

$$\frac{0.800-q}{3.476-q}=-0.0437\;e^{-0.136(t-10)}.$$

The 30 min point is again calculated to be 0.81 meq·g^−1^, in agreement with the original desorption equation.

The same treatment can be applied to interrupted adsorption. Consider the illustration given earlier in connection with table 1. If the adsorption had been interrupted after having been allowed to proceed for, say, 20 min, and the amount adsorbed per gram at that time were considered the new initial conditions; what would the resultant resorption equation become? Again, *M* and *N* would be unchanged, but now *q*_I_ (instead of being zero as at the beginning of the original experiment) would take on the new value of 0.4667. Immediately, the resorption equation in that instance could be written:

$$\frac{0.566-q}{1.130-q}=+0.150\;e^{-0.0604(t-20)}$$

where the coefficient +0.150 is determined from

$$\frac{0.566-q_{I}}{1.130-q_{I}}$$

according to eq (13). The fact that the coefficient 0.150 remains greater than zero shows that the resorption in this case is an adsorption process.

If after 20 min in this same illustration, the solution had been diluted by adding water until its volume, *V*, became, say, 500 ml the situation would have changed considerably. While *q*_I_ would still be 0.4667, the initial concentration *c*_I_ would now become (0.0166)/5 or 0.00333 meq·ml^−1^. The new value of *c*_0_ according to eq (12) would be 0.00613. It would now be possible to recompute *M* from eq (7) and then to redetermine *N* from eq (8). The new values would be *M*=0.947; *N*=0.506. The resorption equation would then reduce to

$$\frac{0.441-q}{1.453-q}=-0.0264\;e^{-0.0217(t-20)}$$

and since the coefficient is now negative, the resumed process would have changed from adsorption to desorption. Clearly, if the degree of dilution had been but slight, the resorption would have continued as an adsorption process but to a diminished extent.

It is instructive to select the final illustration from an experiment cited by Burgers [4] in referring to a paper by Freundlich [6] published nearly 60 years ago. Freundlich compared two batch adsorption runs using 1g of blood charcoal as the adsorbent in each run and acetic acid as the adsorbate. The second run used twice the initial concentration, but only half of the volume. However, after a reasonably long period of time, the second batch was diluted with an equal volume of water and stirring was continued for an additional hour—presumably long enough to re-establish equilibrum. Both runs ended under comparable conditions, yet the final solution concentration was slightly lower in the second experiment than in the first. Freundlich ignored the difference and used the illustration to prove the reversible nature of adsorption. It should be possible in light of the present theory to re-examine the data quantitatively in an attempt to account for the observed discrepancy.

Freundlich’s measurements are shown in table 12. For his first batch, the initial value of *c* was also *c*_0_, since all of the acetic acid was in solution. The final condition corresponded to a *q* value of 0.802 meq·g^−1^ as indicated in the last column of table 12. In his second batch before dilution, *c*_0_ was 0.1376 meq·ml^−1^ while after dilution, it reverted back to 0.06880 meq·ml^−1^. The final concentration of the second batch after dilut ion corresponded to *q*=0.816 meq·g^−1^.

If the present theory applies to Freundlich’s experiment, it ought to be possible to assign reasonable values to the three parameters, *q*_0_, *k*_1_, and *k*_2_, consistent with results already discussed for similar systems under substantially the same conditions. If it is estimated that

$$\begin{array}{l} q_{0}=1.07\;\text{meq}\cdot g^{-1}.\\ k_{1}=30.0\;\text{ml}\cdot {\text{meq}}^{-1}\cdot {\text{hr}}^{-1}(0.5\;\text{ml}\cdot {\text{meq}}^{-1}\cdot \min^{-1})\\ \frac{k_{2}}{k_{1}}=0.02\;\text{meq}\cdot {\text{ml}}^{-1}, \end{array}$$

the sorption equations applicable to both batches are determined as follows:

*For the first batch:*

$$M=\frac{100}{2}\left[0.02+0.06880\left(1.000+\frac{1.00\times 1.07}{100\times 0.06880}\right)\right]=4.975$$

and

$$M^{2}-N^{2}=(1.07)(6.880)=7.362\;\text{so},\;N=4.170$$

hence,

$$\frac{M-N}{M+N}=+0.0880.$$

Finally, the adsorption equation takes the form:

$$\frac{0.805-q}{9.145-q}=0.0880e^{-2.502t}$$

which may be solved for *q* when *t*=20.5 hr to give *q*=0.805 meq·g^−1^ compared with 0.802 in table 12.

*For the second batch—before dilution:*

$$M=\frac{50}{2}\left[0.02+0.1376\left(1.000+\frac{1.00\times 1.07}{50\times 0.1376}\right)\right]=4.475$$

*M*^2^−*N*^2^= (1.07) (6.880) = 7.362, as before; but now

N=3.559

so in this instance $\frac{M-N}{M+N}=0.1140$. Therefore, the adsorption equation applicable to this case becomes:

$$\frac{0.916-q}{8.034-q}=0.1140e^{-4.271t}.$$

This would require that at the time of dilution; namely, when *t*=21.0 hr, the value of *q* would have been 0.916 meq·g^−1^ (although it was not actually measured).

*For the second batch—after dilution:* The addition of 50 ml of water would have the effect on *M* and *N* of causing them to revert back to the values 4.975 and 4.170, respectively, which applied to the first batch. This is evident from the definitions of *M* and of *M*^2^−*N*^2^ in eqs (7) and (8). The only difference is that *q*_I_ would now be 0.916 meq·g^−1^ where originally it was zero. Therefore, the coefficient of the exponential becomes:

$$\frac{0.805-0.916}{9.145-0.916}=-0.0135$$

which, being negative, means desorption. The final resorption equation can therefore be written by inspection:

$$\frac{0.805-q}{9.145-q}=-0.0135e^{-2.502t}.$$

The final condition after dilution and resorption was reached 1 hr later. By substitution of *t*=1.0 in this equation, it is found that *q*=0.814 meq·g^−1^ which is in good agreement with 0.816 in table 12.

## 7. Summary

Batch adsorption from solution can be characterized and interpreted in terms of the parameters *q*_0_, *k*_1_ and *k*_2_ whose values best fit the general adsorption equation, eq (6).

In the special case where the desorption rate constant can be neglected, a simplified two-parameter equation is adequate for adsorption. Short-cut methods have been found for evaluating the parameters from the data:

- when the adsorption is a function of time or
- when the adsorption is a function of both *W/V* and *c*_0_.

Values of the parameters can also be determined for the general case where the adsorption data are time dependent.

The characteristic parameters determined from batch adsorption are in agreement with corresponding values determined from column adsorption.

Some deviations in the results have been observed in certain instances and can be explained in terms of a slight departure from the theoretical model.

Even the general adsorption equation, eq (6), can be considered as a special case of eq (13) which, differing only in initial conditions but utilizing the same set of parameters, will, in fact, predict with equal facility desorption, interrupted sorption, and sequential combinations of adsorption and desorption as the case may be.

## Figures and Tables

**Table 1:** Data of batch adsorption Run #201

(Drvden and Kav)	
Acetic acid: *V* =100 ml *c*_0_=0.0306 meq*·*ml^−1^	Coconut charcoal: *W*=3.0 g Sieve size: (8 on 10)
Temperature : 30 °C Stirring rate: 400 rpm	

*t*	c	*q*
		
*min*	*meq·ml^−^*^1^	*meq·g^−^*^1^
…..	…..	…..
10.0	0.0200	0.3533
15.0	.0185	.4033
20.0	.0166	.4667
30.0	.0155	.5033
45.0	.0147	.5300
60.0	.0140	.5533

**Table 2:** Batch adsorption data for acetic acid on “Norit” (W. G. Burgers) Each determination was carried out at 25 °C with *V*=50 cm^3^ and *t*=2.0 hr, but with initial concentrations and adsorbent weights as indicated.

C_0_	*q*(obs)		
	*W*=1.0 g	*W*=2.0 g	*W*=3.0 g
			
*mM*·*cm*^−3^	*mM*·*g^−^*^1^	*mM*·*g*^−1^	*mM*·*g^−^*^1^
0.0106	0.354	0.224	…..
.0323	.725	.551	…..
.0489	.908	.737	…..
.0546	…..	…..	0. 655
.0643	1.030	.876	…..
.0881	1.199	…..	…..
.0917	1.240	1.124	…..
.1091	…..	…..	1.056
.1796	1.654	1.514	1.396
.2188	1.777	1.646	
. 2588	1.869	1.750	1.671
.3146	1.957	1.896	…..
.3779	2.082	2. 024	1.950

**Table 3:** Adsorption calculations from the data of table 2 Estimated values of 2.1, 6.0, and 0.02 for the parameters *q*_0_, *k*_1_, and *k*_2_/*k*_1_, respectively, were used in the fitting of eq (6).

c_0_	*W/V*	*M*^2^*−N*^2^	*M*	*M−N*	*M+N*	$2\left(\frac{W}{V}\right)Nk_{1}t$	*q*_(calc)_	*q*_(obs)_
								
*mM·cm*^−3^	*g·cm*^−3^						*mM·g*^−1^	*mM·g*^−1^
0.2588	0.060	9.0573	3.3869	1.8333	4.9405	2.237	1.60	1.671
.1796	.060	6.285	2.713	1.677	3.749	1.492	1.45	1.396
.3779	.060	13.2258	4.3643	1.951	6.777	3.475	1.91	1.950
.0643	.040	3.3758	2.103	1.079	3.127	0.983	0.78	0.876
.3146	.040	16.517	5.232	1.937	8.527	3.163	1.87	1.896
.2188	.040	11.487	4.035	1.845	6.225	2.102	1.68	1.646
.0489	.020	5.1345	2.773	1.175	4.371	0.767	0.72	0.908
.2188	.020	22.974	7.020	1.891	12.149	2.462	1.75	1.777
.0881	.020	9.251	3.752	1.554	5.950	1.055	1.11	1.199

**Table 4:** Characteristic parameters determined from column adsorption experiments Results are based on the static-bed data (C. E. Dryden) for acetic acid on coconut charcoal at 30 °C fitted to eq (11).

Run No.	Sieve size	*c*_0_	$\dot{V}$	*x*	*q*_0_	*k*_1_
						
		*meq*·*ml^−^*^1^	*ml*·*min^−^*^1^	*q*	*meq*·*g^−^*^1^	*ml*·*meq^−^*^1^·*min^−^*^1^
18	(8–10)	0.10	21.8	59.8	0.850	0.769
19	(8–10)	.10	35.0	59.2	.676	.575
20	(8–10)	.10	6.99	18.8	.928	.677
21	(8–10)	.10	3.32	18.9	1.176	.379
36	(8–10)	.10	4.88	59.4	1.333	.398
53	(12–16)	.10	9.18	18.1	1.259	1.124
55	(12–16)	.10	3.49	18.5	1.330	0.867
56	(12–16)	.10	22.9	18.4	1.023	1.845
57	(12–16)	.10	23.2	59.1	1.363	1.259
58	(12–16)	.10	64.2	59.1	1.057	2.259
60	(12–16)	.31	3.81	17.9	2.100	0.664
61	(12–16)	.31	45.4	60.6	1.279	.690
75	(24–30)	.10	37.7	55.6	1.542	2.798
76	(24–30)	.10	57.8	17.0	1.336	5.682
77	(24–30)	.10	25.2	17.3	1.625	3.059
78	(24–30)	.10	9.53	16.9	1.583	2.628
79	(24–30)	.10	64.6	55.5	1.358	3.115
83	(40–60)	.10	13.9	15.4	1.470	11.569
84	(40–60)	.10	66.6	15.5	1.406	22.33
90	(40–60)	.10	25.3	15.6	1.443	9.673
91	(40–60)	.31	13.1	15.6	2.210	3.642
92	(40–60)	.31	63.6	15.5	1.935	5.697

**Table 5:** Data of batch adsorption Run #221

(C. E. Dryden)	
Acetic acid : *V*=100 ml *c*_0_=0.0308 meq·ml^−1^	Coconut charcoal: *W*=10.0 g Sieve size: (8 on 10)
Temperature: 30 °C Stirring rate: 400 rpm	

*t*	*c*	*q*
		
*min*	*meg*·*ml*^−1^	*meq*·*g*^−1^
…..	…..	…..
10.0	0.0100	0.206
15.0	.0084	.222
20.0	.0075	.231
30.0	.0061	.245
45.0	.0053	.253
60.0	.0048	.258
75.0	.0044	.262
∞	.0040	.266

**Table 6:** Batch comparisons: the consequences of prewetting and the effect of varying the amount of adsorbent

Sieve size: (8 on 10)	Stirring rate: 400 rpm
Temperature: 30 °C	

Run No.	Initial state	*W*	*V*	*c*_0_	*q*_0_	*k*_1_
						
		*g*	*ml*	*meq*·*ml*^−1^	*meq*·*g*^−1^	*ml*·*meq*^−1^·*min*^−1^
222	dry	1.0	100	0.03	0.726	2.19
236	wet	2.48	108	.027	.594	3.06
201	dry	3.0	100	.03	.565	3.74
238	wet	4.01	107	.026	.474	4.45
220	dry	5.0	100	.03	.421	5.26
221	dry	10.0	100	.03	.262	8.50

**Table 7:** Batch comparison: effect of W/V

V=100 ml *c*_0_=0.03 meq·ml^−1^		Stirring rate: 400 rpm Sieve size: (12 on 16)
	Temperature: 30 °C	

Run No.	*W*	*q*_0_	*k*_1_
			
	*g*	*meq*·*g*^−1^	*ml*·*meq*^−1^·*min*^−1^
217-8-9	1.0	0.672	2.64
206	3.0	.562	4.77
210	5.0	.462	5.19
216	10.0	.271	15.9

**Table 8:** Batch comparison: effect of stirring rate

*V*=100 ml *c*_0_=0.03 meq·ml^–1^	*W*=3.0 g Temperature: 30 °C

Run No.	Stirring rate	*q*_0_	*k*_1_

8–a Sieve size: (12 on 16)			

	*rpm*	*meq*·*g*^−1^	*ml*·*meq*^−1^·*min*^−1^
208	0	0.522	2.97
207	120	.541	6.16
206	400	.562	4.77

8–b Sieve size: (8 on 10)			

203	0	0.590	1.26
209	150	.498	14.6
201	400	.565	3.74

**Table 9:** Batch comparison: effect of c_0_

*V*=100 ml		Stirring rate: 400 rpm
	Temperature: 30 °C	

Run No.	*c*_0_	*q*^0^	*k*_1_

*W*=3.0 g	9–a		Sieve size: (8 on 10)

	*meq*·*ml*^−1^	*meq*·*g*^−1^	*ml*·*meq*^−1^·*min*^−1^
201	0.03	0.57	3.74
202	.10	1.425	0. 819

*W*=5.0 g	9–b		Sieve size: (12 on 16)

210	0.03	0.462	5.19
215	.10	1.201	0.905

**Table 10:** Batch comparison: effect of particle size

*c*_0_=0.03 meq·ml^−1^	Stirring rate: 400 rpm
Temperature: 30°C	

Run No.	Sieve size	*q*^0^	*k*_1_
			
	10–a	*W*= 1.0g	

		*Meq*·*g*^−1^	ml·*meq*^−1^·*min*^−1^
222	(8 on 10)	0.726	2.19
217-8-9	(12 on 16)	.672	2.64
			
	10–b	*W*=3.0 g	

201	(8 on 10)	0.565	3.74
206	(12 on 16)	.562	4.77
212	(24 on 30)	.608	15.22
213	(40 on 60)	.567	71.5
214	(80 on 100)	.653	110.5

	10–c	*W*=5.0 *g*	

220	(8 on 10)	0.421	5.26
210	(12 on 16)	.462	5.19

	10–d	*W*= 10.0 g	

221	(8 on 10)	0.262	8.50
216	(12 on 16)	.271	15.9

**Table 11:** Data of batch desorption Run #224

(C. E. Dryden)		
Acetic acid: Stirring rate: 400 rpm		Coconut charcoal: Sieve size: (8 on 10)
	Temperature: 30 °C	

*t*	*c*	*q*
		
*min*	*meq*·*ml*^−1^	*meq*·*g*^−1^
1.0	0.0102	1.190
2.0	.014	1.115
3.0	.0169	1.058
4.0	.0188	1.020
5.0	.0204	0.989
7.5	.0221	.955
10.0	.0243	.912
15.0	.0266	.867
20.0	.0275	.849
30.0	.0282	.835
45.0	.0293	.814
60.0	.0299	.802

**Table 12:** Freundlich’s experiment

	*W*	*V*	*t*	*c*	*q*
					
	*g*	*ml*	*hr*	*meq*·*ml*^−1^	*meq*·*g*^−1^
First batch					
Initial state	1.0	100.0	0	0.06880	0
Final state	1.0	100.0	20.5	.06078	0.802
Second batch					
Before dilution:					
Initial state	1.0	50.0	0	.1376	0
Final state	1.0	50.0	21.0		( )
After dilution:					
Initial state	1.0	100.0	21.0		( )
Final state	1.0	100.0	22.0	.0064	0.816

## Footnotes

1 Figures in brackets indicate the literature references at the end of this paper.

2 Since the three independent parameters must be evaluated from the experimental points, no less than three points are required. The use of four points affords a degree of flexibility in the choice of data. The only restriction is that *i ≠ j*, although it is permissible for 2*i=j.* In the latter instance, the minimum of three points would, of course, result.
